# Caspase-1 Inhibitor Reduces Pyroptosis Induced by Brain Death in Kidney

**DOI:** 10.3389/fsurg.2021.760989

**Published:** 2021-11-26

**Authors:** Weifeng Liu, Dongjing Yang, Jihua Shi, Peihao Wen, Jiakai Zhang, Zhihui Wang, Bowen Hu, Xiaoyi Shi, Shengli Cao, Wenzhi Guo, Shuijun Zhang

**Affiliations:** ^1^Department of Hepatobiliary and Pancreatic Surgery, The First Affiliated Hospital of Zhengzhou University, Zhengzhou, China; ^2^Henan Engineering Technology Research Center of Organ Transplantation, Zhengzhou, China; ^3^Zheng Zhou Key Laboratory of Hepatobiliary and Pancreatic Diseases and Organ Transplantation, Zhengzhou, China; ^4^Department of Hepatobiliary and Pancreatic Surgery, The First Affiliated Hospital and College of Clinical Medicine, Henan University of Science and Technology, Luoyang, China

**Keywords:** brain death, renal injury, pyroptosis, caspase-1, caspase-11, hypoxia

## Abstract

Brain death (BD) induces an organ-level inflammatory response. However, the underlying mechanisms have not been fully elucidated. Here, we investigated the role of caspase-1-mediated pyroptosis in BD-induced kidney injury in rats. A BD model was established in Sprague-Dawley rats. The rats were intravenously injected with Z-YVAD-FMK 1 h before BD, and sham-operated rats served as controls. After 0, 1, 2, 4, and 6 h of BD, renal injury, and renal expression of the nod-like receptor family pyrin domain-containing 3 (NLRP3), caspase-1, caspase-11, gasdermin D (GSDMD), IL-1β, and IL-18 were assessed using quantitative reverse transcriptase-polymerase chain reaction, western blotting, and immunohistochemistry. Blood urea nitrogen and serum creatinine levels were measured. Additionally, renal tubular epithelial cells (NRK-52E) were subjected to 3 h of hypoxia followed by 6 h of reoxygenation and incubated with Z-YVAD-FMK before hypoxia and reoxygenation. Caspase-11 was knocked-down using small interfering RNA technology. Cell viability and levels of pyroptosis-associated proteins were assessed thereafter. NLRP3, caspase-1, GSDMD, IL-1β, and IL-18 expression levels were upregulated in BD rats. Treatment with Z-YVAD-FMK reduced mRNA and protein levels of caspase-1, GSDMD, IL-1β, and IL-18, improved renal function, and alleviated renal injury. Z-YVAD-FMK efficaciously reduced pyroptosis effects in kidneys in BD rats. Thus, it could be considered as a therapeutic target for BD-induced kidney injury.

## Introduction

Brain death (BD) induces organ injury in donors by stimulating an inflammatory response (1, 2). Many retrospective analyses and randomized controlled studies have confirmed that kidneys derived from brain-dead organ donors show inferior survival and delayed functional recovery than those derived from living donors (3, 4). However, the mechanism underlying the effect of BD on donor organ function has not been fully elucidated. Brain-dead donors show inflammatory responses at the organ level, and the degree of response is related to the extent of organ dysfunction after transplantation (5, 6).

The kidney is particularly sensitive to ischemia and hypoxia, and cell death in kidney diseases have been studied primarily in the context of tubular injury. The renal tubule is the key site of BD-associated injury and an important source of inflammatory cytokines (7, 8). Renal cell death is a core pathophysiological factor in any renal disease (9). Thus, exploring the mechanisms of cell death and tissue damage can provide major insights for disease treatment.

Pyroptosis is a highly specific type of inflammatory programmed cell death that differs from necrosis and apoptosis (10). Pyroptosis is activated by caspase-1 (human and mouse), caspase-4 and caspase-5 (human), or caspase-11 (mouse) (11). Ge et al. (12) identified the pathological roles of NLRs and AIM2 inflammasomes in the damaged blood-brain barrier after traumatic brain injury. NLRP3 promotes the activation of caspase-1, induces release of IL-1β and IL-18, and leads to tissue injury (13). GSDMD, a substrate of both caspase-1 and caspase-11/4/5, is primarily expressed in immune cells and shows unique structural characteristics of a perforating protein (14). Caspase-1 inhibitor Ac-YVAD-CMK inhibits pyroptosis in brain microvascular endothelial cells (12). The role of inflammasome and caspase-1 activation pathway in stroke and traumatic brain injury has been previously confirmed (15, 16).

Yang et al. (17) found pyroptosis of renal tubular epithelial cells to be the key event in mouse kidney ischemia-reperfusion injury. Meanwhile, Wang et al. (18) found renal injury and pyroptosis to be typical events after renal ischemia-reperfusion. However, whether the canonical and non-canonical pyroptosis pathway mediates renal injury following BD remains unclear.

Although pyroptosis has gained attention in the field of inflammation, research on organ injury-related pyroptosis in brain-dead donors remains insufficient. Therefore, we aimed to investigate the role of caspase-1-mediated pyroptosis in BD-induced rat kidney injury, and the effect of a caspase-1 inhibitor (Z-YVAD-FMK) on it to reveal potential target genes for future intervention.

## Materials and Methods

### Experimental Animals

Male Sprague-Dawley rats, weighing 250–300 g, were purchased from the Animal Center of the Medical College of Zhengzhou University. The rats were housed at 18–22 °C, with moderate humidity, 12 h light/dark cycle, and a quiet environment. They were allowed access to chow and drink ad libitum. All experiments were approved by the Ethics Committee of the First Affiliated Hospital of Zhengzhou University (No. 2019-KY-019).

### Animal Groups and Treatment

The BD rat model was established based on previous studies in our laboratory (19) and available literature (20–22). A total of *n* = 72 rats were used for the experiments. Rats were randomly categorized into two cohorts, control cohort (*n* = 36) and Z-YVAD-FMK-treated cohort (Z cohort) (*n* = 36). In each cohort, rats were randomly categorized into sham, 0, 1, 2, 4 and 6h group (*n* = 6 for each group). Rats were fasted for 6 h before the experiment but allowed free access to water. BD was induced by inflating a subdural balloon catheter to induce slow and intermittent intracranial compression. Blood samples from the abdominal aorta and kidney tissues were collected at 0, 1, 2, 4, 6 h after BD and sham group in each cohort. Operations in sham group were performed in the same manner as that in the BD group, but BD was not established. Z-YVAD-FMK (Abmole Bioscience Inc., Shanghai, China) was dissolved in 14 μL of dimethyl sulfoxide (DMSO) and intravenously administered at 300 ng/kg 1 h before BD. Rats were euthanized under general anesthesia and were sacrificed at different time points as indicated. The abdominal cavity was opened, kidney was removed, and macroscopic ischemia, necrosis, and other lesions were visually observed. Lower portion of the right kidney was collected and fixed in 4% paraformaldehyde solution, followed by paraffin embedding, sectioning, and hematoxylin and eosin staining. Paller score was used to evaluate pathological changes in the kidney by pathologist (23). The standard is described as follow: normal renal tubules (0 point), obviously dilated renal tubules (1 point), flat or swollen cells are scored (1 point), renal brush border membrane injury (1 point), cell debris (2 points), tubular type (2 points), cell shedding and necrosis in the lumen of renal tubules but without tubular type and cell debris (1 point) (24). Expression of caspase-1 and caspase-11 in renal tissues was determined by immunohistochemistry (IHC).

### Cell Culture and Treatment

NRK-52E cells (rat ductal epithelial cells; Procell Life Science & Technology Co., Ltd., Wuhan, China) were cultured in Dulbecco's modified Eagle's medium (Solarbio, Beijing, China) with 10% fetal bovine serum (Gibco, Gaithersburg, MD, USA) and 100 U/ml penicillin-streptomycin (Solarbio, Beijing, China) in 37°C incubator with 5% CO_2_.Cell experiment were categorized into four groups: normal control, hypoxia/reoxygenation (H/R), Z-YVAD-FMK, and DMSO control. Before the experiment the medium was changed into serum free medium for 8 h. In the normal control group, cells were incubated in 5% CO_2_ and 1% O_2_ at 37 °C for 3 h. In the H/R group, the culture dishes were incubated in 5% CO_2_ and 1% O_2_ at 37 °C for 3 h (BD-induced hypoxia stimulation) (25). Then, the cells were reoxygenated for 2, 4, 6, 8, and 12 h. In the Z-YVAD-FMK group, Z-YVAD-FMK was added into the medium 1 h before H/R at different concentrations (5, 10, 25, and 50 μM; DMSO volume = 80 μL), and their effects on hypoxia-stimulated NRK-52E cells were detected. Finally, in the DMSO control group, 80 μL DMSO was added to each dish 1 h before experiment, and the cells were incubated in 5% CO_2_ and 1% O_2_ at 37 °C for 3 h. Each experiment was repeated thrice.

Next, NRK-52E cells were transfected with small interfering RNA (siRNA) or negative control (NC) and threated with hypoxia/reoxygenation conditions. Three SiRNA were used to decrease the expression of caspase-11. The si-caspase-11 and si-NC sequences were as follows: Si-RNA-1: sense 5′-GGGCAACCUUGACAAGAUATT-3, Antisense 5′-UAUCUUGUCAAGGUUGCCCTT-3′, Si-RNA-2: sense 5′-GCUCUUAUCAUAUGCAAUATT-3, Antisense 5′-UAUUGCAUAUGAUAAGAGCTT-3′, Si-RNA-3: sense 5′-CUCCAGAUGUGCUAUUAUATT, Antisense UAUAAUAGCACAUCUGGAGTT-3′, Negative control(NC): sense 5′-UUCUCCGAACGUGUCACGUTT-3, Antisense 5′-ACGUGACACGUUCGGAGAATT-3. Finally, we harvested the cells, extracted RNA, reverse transcribed RNA into cDNA, and extracted cell proteins as previously described (25). Transfection effects of siRNA were determined by reverse transcription-quantitative polymerase chain reaction (RT-qPCR) and western blotting.

### Cell Viability Check by Cell Counting Kit-8 (CCK-8)

NRK-52E cell suspension was seeded in 96-well plates at 100 μL/well, and pre-cultured at 37 °C and 5% CO_2_. After treating the cells as described above, CCK-8 reagent (10 μL) was added to each well and incubated for 1 h. Absorbance was recorded at 450 nm. Each experiment was repeated thrice.

### Reverse Transcription-Quantitative Polymerase Chain Reaction (RT-QPCR)

The mRNA levels of NLRP3, caspase-1, caspase-11, IL-1β, and IL-18 were measured by RT-qPCR. Total RNA was extracted from tissues using the TRIzol (Thermo Fisher Scientific, Shanghai, China), and reverse transcription was performed as described previously (26). Primers were designed based on the gene sequences acquired from PubMed. The primers were synthesized by Invitrogen (Shanghai) Trading Co., Ltd., China, and are shown in Table 1. The primers were diluted appropriately, PCR amplification was performed, and RT-qPCR results were analyzed using a relative quantitative method as described previously (26).

**Table 1:** Gene-specific quantitative-polymerase chain reaction primers.

**Primer**	**Primer sequence**
Nlrp3-f	5′-CTGCATGCCGTATCTGGTTG-3′
Nlrp3-r	5′-GCTGAGCAAGCTAAAGGCTTC-3′
Casp1-f	5′-GACCGAGTGGTTCCCTCAAG-3′
Casp1-r	5′-GACGTGTACGAGTGGGTGTT-3′
Casp11-f	5′-CAGGAGCCCACTCCTACAGA-3′
Casp11-r	5′-AGGACAAGTGGTGTGGTGTT-3′
GSDMD-f	5′-AAGATCGTGGATCATGCCGT-3′
GSDMD-r	5′-AACGGGGTTTCCAGAACCAT-3′
IL-1β-f	5′-AGGCTGACAGACCCCAAAAG-3′
IL-1β-r	5′-CTCCACGGGCAAGACATAGG-3′
IL-18-f	5′-ACCACTTTGGCAGACTTCACT-3′
IL-18-r	5′-GGATTCGTTGGCTGTTCGGT-3′
Acta2-f	5′-CCGAGATCTCACCGACTACCTCA-3′
Acta2-r	5′-TCAAAGTCCAGAGCGACATAGCA-3′

### Western Blot Analysis

Cell and tissue proteins were extracted as previously described (19), and bicinchoninic acid method was used to determine the protein concentration. Nitrocellulose membranes were incubated with primary antibodies (anti-NLRP3, anti-IL-18, GAPDH, Proteintech Group, Inc., Chicago, IL,USA; anti-GSDMD, Abbexa Ltd, Cambridge, United Kingdom; anti-caspase-1, anti-IL-1β, anti-cleaved caspase-1, anti-cleaved IL-1β, Affinity Biosciences, Cincinnati, OH, USA; anti-caspase-11 p20, Santa Cruz Biotechnology, Inc.Dallas, Texas, USA) at 4 °C overnight. The membranes were washed with 1% TBST before and after incubation with goat anti-rabbit IgG secondary antibody (LI-COR Biotechnology, Lincoln, NE, USA) or goat anti-mouse IgG secondary antibody (LI-COR Biotechnology, Lincoln, NE, USA) for 1 h at room temperature. Odyssey CLx imaging system (LI-COR Biosciences, Lincoln, NE, USA) was used to analysis protein expression as previously described (19).

### Biochemical Determination

Blood samples extracted from the abdominal aorta were centrifuged at 10,000 *g* for 20 min at 4°C. Frozen serum in the upper layer was collected, and serum creatinine (Cr) and urea nitrogen levels were measured using a Commercial Kit (Jiancheng Biotech, Nanjing, China) following the manufacturer's instructions.

### Statistical Analysis

SPSS 19.0 (SPSS Inc., Chicago, USA) was used for statistical analysis. Student's *t*-test was used to calculate the difference between the data obtained from two groups. One-way analysis of variance was used to calculate the difference across the data of multiple groups. Results are expressed as mean ± standard deviation. Results with *P*-values < 0.05 were considered as statistically significant.

## Results

### Pyroptosis Occurring in BD Rats Promoted Inflammation and Induced Kidney Injury

**Figure 1:**
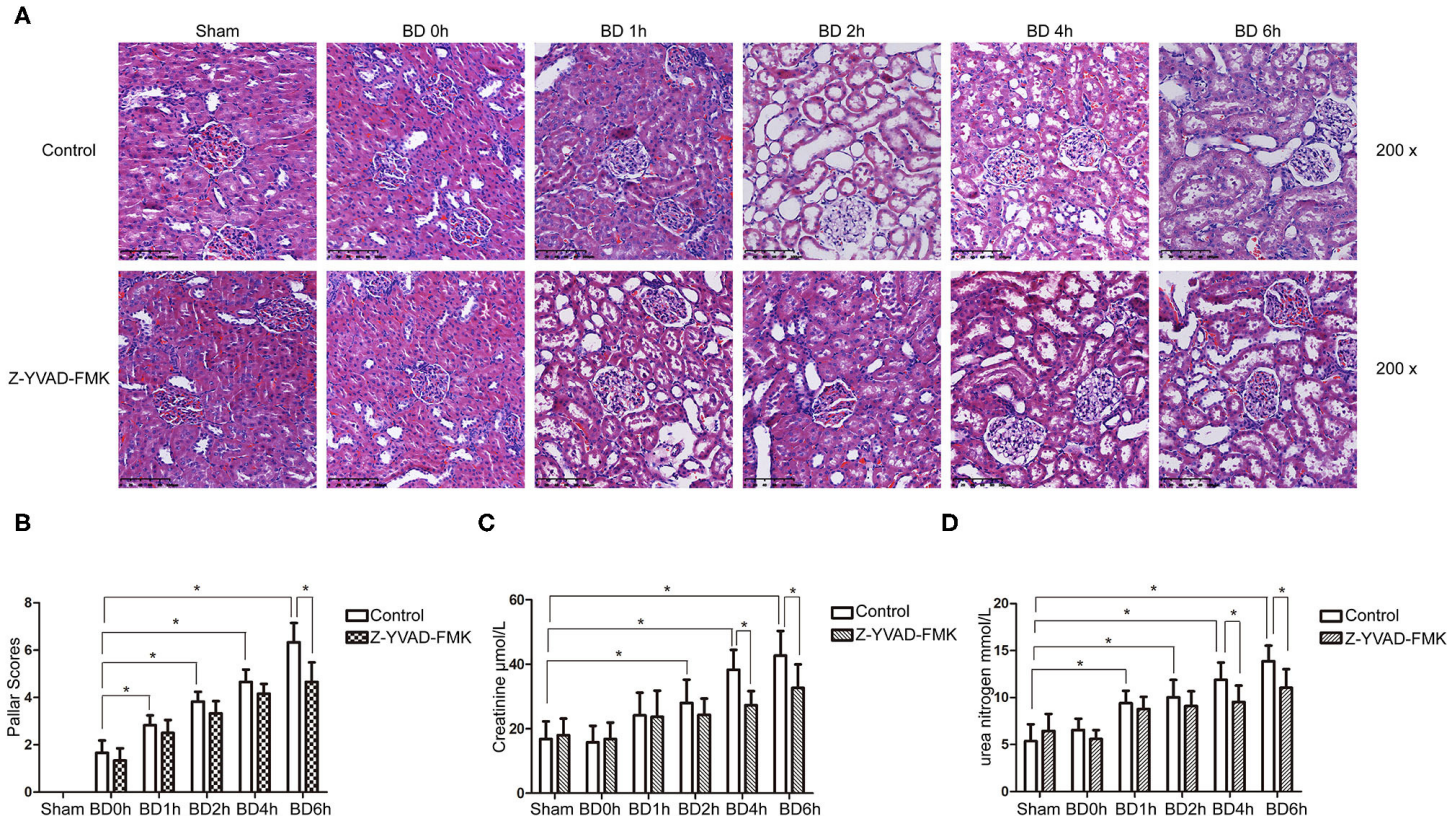
HE staining, Paller scores and renal function results in control cohort and Z-YVAD-FMK-treated cohort. There were sham, 0, 1, 2, 4, and 6 h group (*n* = 6 in each group) in each cohort. HE appearance after **(A)**. Paller score **(B)**. Serum creatinine levels **(C)**. Urea levels **(D)**. *p < 0.05. BD = brain-death; Sham = without brain death; HE = Hematoxylin and eosin.

**Figure 2:**
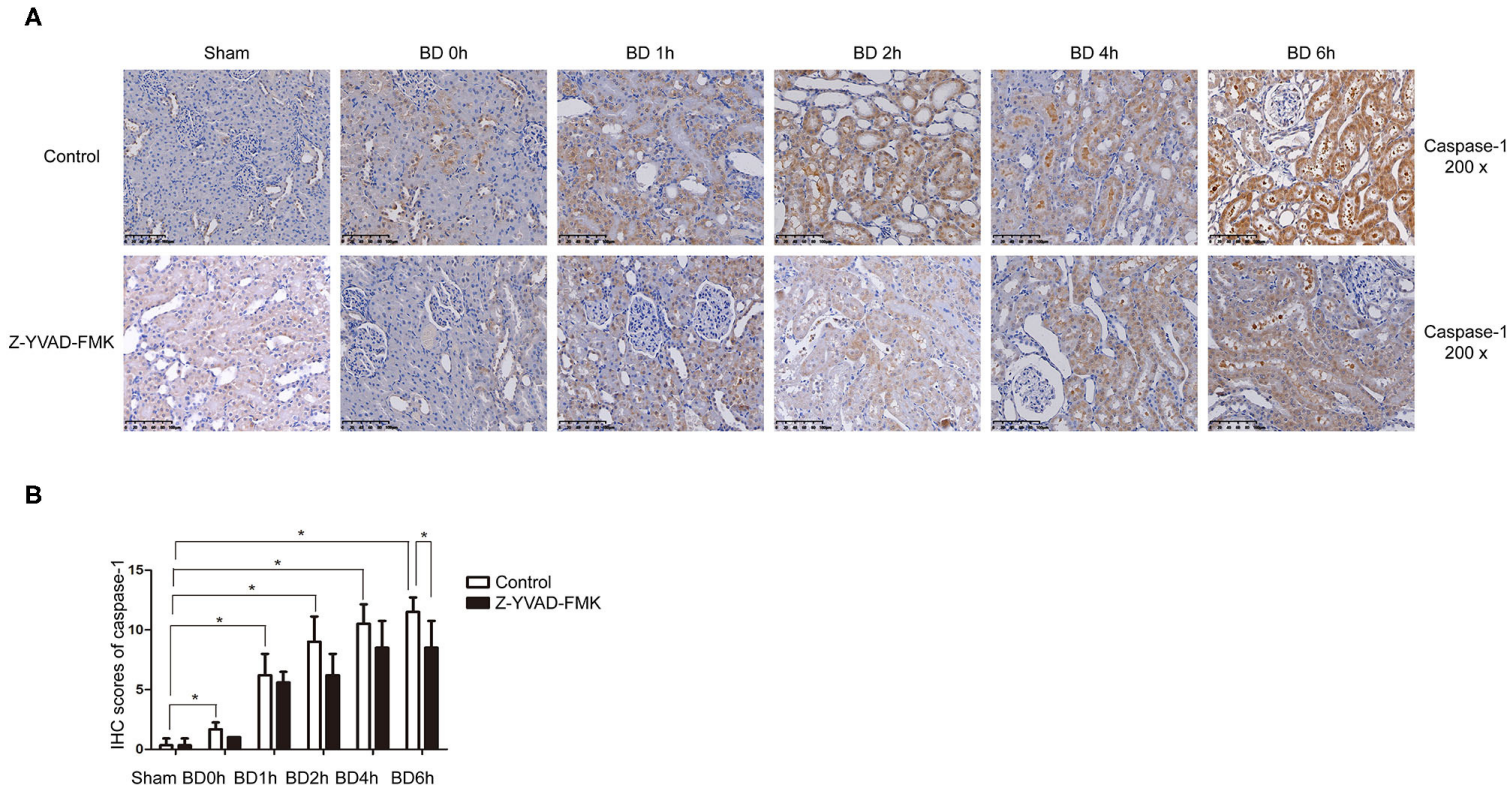
Immunohistochemical (IHC) score of caspase-1 in control cohort and Z-YVAD-FMK-treated cohort. There were sham, BD 0, 1, 2, 4, and 6 h group (*n* = 6 in each group) in each cohort. (Magnification 200×). **(A)** Control cohort and Z-YVAD-FMK-treated cohort. **(B)** IHC score of caspase-1 in different cohorts. *p < 0.05. BD = brain-death; Sham = without brain death.

**Figure 3:**
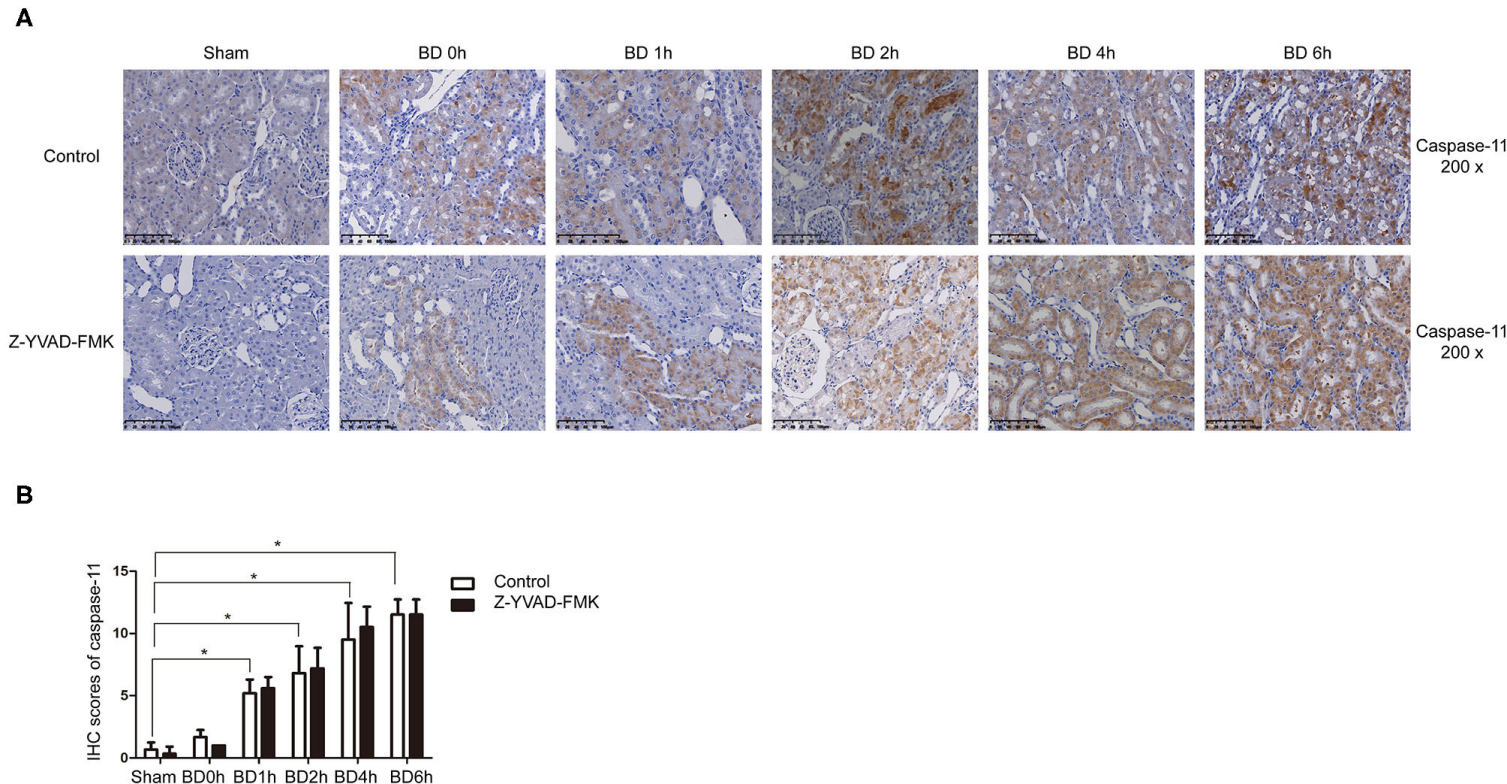
Immunohistochemical (IHC) score of caspase-11 in control cohort and Z-YVAD-FMK-treated cohort. There were sham, BD 0, 1, 2, 4, and 6 h group (*n* = 6 in each group) in each cohort. (Magnification 200×). **(A)** Control cohort and Z-YVAD-FMK-treated cohort. **(B)** IHC score of caspase-11 in different groups. *p < 0.05. BD = brain-death; Sham = without brain death.

As can be seen in Figure 1A, proportion of necrotic renal tubular cells increased after brain death. Paller scores in the BD 1, 2, 4, and 6 h group were higher than BD 0 h group (Figure 1B). In the BD + Z-YVAD-FMK group, Paller scores were reduced than those in the control group at 6 h (Figure 1B). The levels of creatinine and urea nitrogen was increased after brain death compared with the sham group (Figures 1C,D). At the 4 and 6 h after brain death, the levels of creatinine and urea nitrogen was significantly lower in Z-YVAD-FMK-treated cohort than control cohort (Figures 1C,D). The proportion of positive cells with caspase-1 and caspase-11 staining increased after brain death (Figures 2, 3). At the 6 h after brain death, the IHC score of caspase-1 was significantly lower in Z-YVAD-FMK-treated cohort than control cohort (Figures 2A,B). However, the IHC score of caspase-11 was not decreased in Z-YVAD-FMK-treated cohort compared with control cohort (Figures 3A,B).

### Effects of Z-YVAD-FMK on MRNA and Protein Expression of Pyroptosis-Related Molecules in Brain-Dead Rats

**Figure 4:**
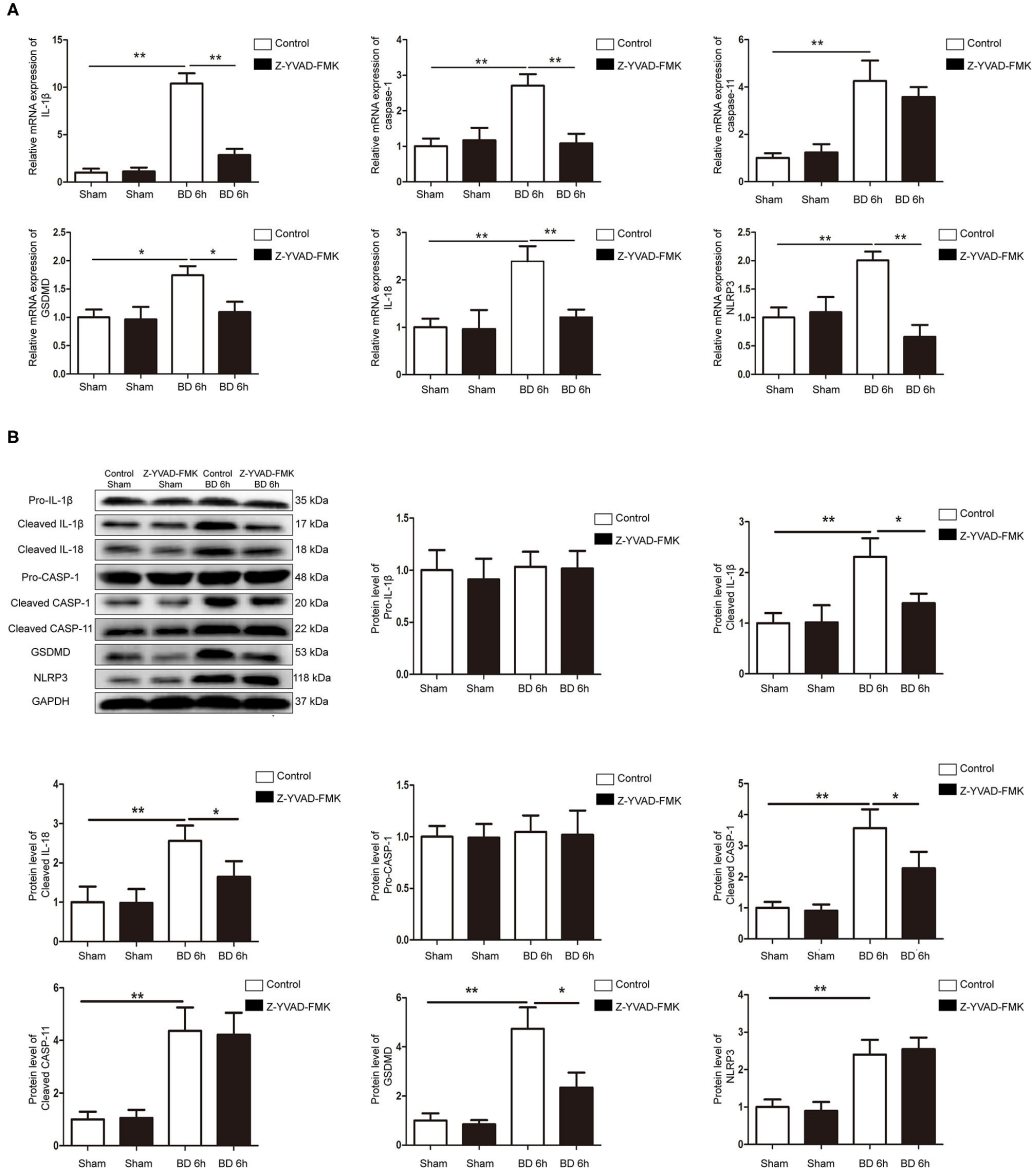
Protein and mRNA expression of pyroptosis-related molecules in control cohort and Z-YVAD-FMK-treated cohort. **(A)** mRNA expression of NLRP3, caspase-1, caspase-11, GSDMD, IL-1β, and IL-18 in rat kidney tissuses. **(B)** Western blotting results of NLRP3, pro-caspase-1, cleaved-caspase-1, cleaved-caspase-11, GSDMD, pro-IL-1β, cleaved-IL-1β and cleaved-IL-18. In rat kidney tissues. *p < 0.05, **p < 0.01. (*n* = 6 in each group). BD = brain-death; Sham = without brain death. GSDMD, gasdermin D; GAPDH, glyceraldehyde-3-phosphate dehydrogenase.

RT-qPCR and western blotting results showed that the expression of NLRP3, caspase-1, caspase-11, GSDMD, IL-1β, and IL-18 at 6 h group were significantly higher compared with sham group in control cohort (Figures 4A,B). However, Z-YVAD-FMK treatment reduced the mRNA and protein levels of caspase-1, GSDMD, IL-1β, and IL-18 in the 6 h group in Z-YVAD-FMK-treated cohort (Figures 4A,B), although caspase-11 expression remained unchanged (Figures 4A,B). The expression of mRNA level of NLRP3 in the 6 h group was significantly lower in Z-YVAD-FMK-treated cohort compared with control cohort (Figure 4A). However, the expression of protein level remained unchanged (Figure 4B).

### Decreasing of Caspase-1 and Caspase-11 Affect the Cell Activity of NRK-52E Cells After H/R

**Figure 5:**
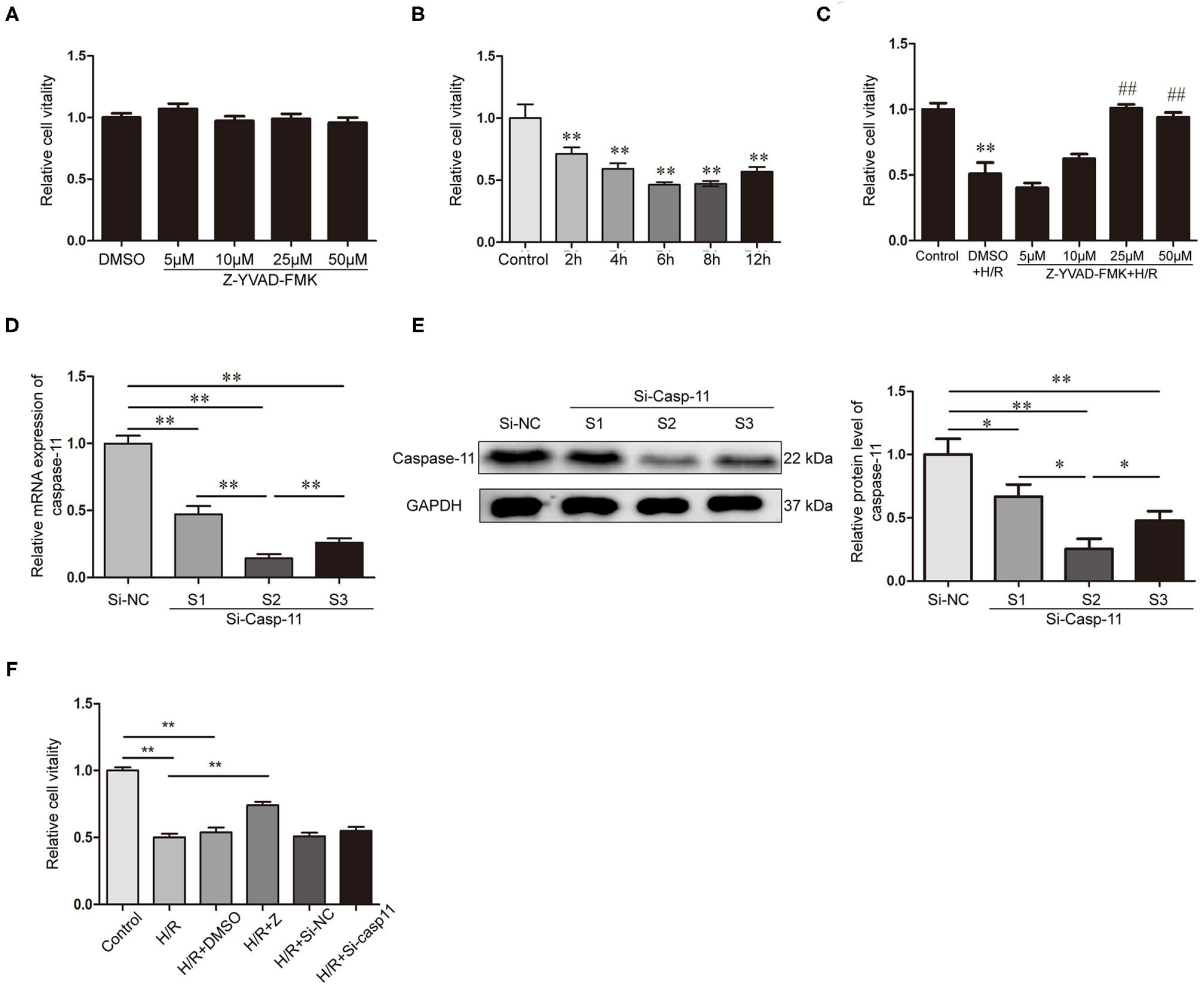
Decreasing of caspase-1 and caspase-11 affect the cell activity of NRK-52E cells after H/R. **(A)** Effects of different concentrations of Z-YVAD-FMK on cell activity in normoxic state. **(B)** NRK-52E cell activity at different times of reoxygenation. **(C)** Effects of different concentrations of Z-YVAD-FMK on the cell activity after 6 h of H/R. **(D)** Expression of caspase-11 mRNA after caspase-11 siRNA transfection. **(E)** Expression of caspase-11 protein and statistical analysis after caspase-11 siRNA transfection. **(F)** Effect of caspase-1 inhibition by Z-YVAD-FMK and caspase-11 inhibition by siRNA on cell activity, as detected by CCK-8. *p < 0.05, **p < 0.01, ^##^*p* < 0.05. Z = Z-YVAD-FMK; H/R, hypoxia/reoxygenation; R, reoxygenation; DMSO, dimethyl sulfoxide; Si-casp11, small interfering ribonucleic acid caspase-11; Si-NC, si-caspase-11 negative control; GAPDH, glyceraldehyde-phosphate dehydrogenase.

NRK-52E cells treated with different concentrations of Z-YVAD-FMK for 12 h. There was no significant difference in the cell activity between the DMSO and Z-YVAD-FMK groups (Figure 5A), indicating that Z-YVAD-FMK had no toxic effect on NRK-52E cells at a concentration of 50 μM (dissolved in DMSO). Cell activity decreased significantly in H/R condition and compared with that in the normal-oxygen group (Figure 5B). The cell activity was lowest at 6 h after reoxygenation (Figure 5B), and we chose this time point for our cell experiment.

Next, we analyzed the activity of NRK-52E cells treated with different concentrations of Z-YVAD-FMK in the H/R environment. Cell viability in the 25 μM and 50 μM Z-YVAD-FMK-treated groups was significantly higher than that in the DMSO-treated group (Figure 5C). However, treatment with 25 μM and 50 μM showed no significant differences (Figure 5C). Therefore, 25 μM Z-YVAD-FMK was used for subsequent experiments.

Transfection effects of siRNA were determined by RT-qPCR (Figure 5D) and western blotting (Figure 5E). Caspase-11 expression was downregulated in NRK-52E cells transfected with siRNA-1, siRNA-2, and siRNA-3 (*p* < 0.01), and its expression was lower in siRNA-2-transfected group than in the siRNA-1 and siRNA-3-transfected groups (Figures 5D,E). Therefore, siRNA-2 was selected for subsequent experiments.

CCK-8 results revealed that NRK-52E cell viability was significantly decreased in the H/R environment and significantly increased upon Z-YVAD-FMK treatment (Figure 5F). However, caspase-11 knockdown with siRNA did not exhibit a protective effect on cell viability after H/R by inhibiting the non-classical pathway of pyroptosis (Figure 5F).

### Protein and MRNA Expression in NRK-52E Cells After Inhibition of Caspase-1 or Caspase-11 in H/R Environment

**Figure 6:**
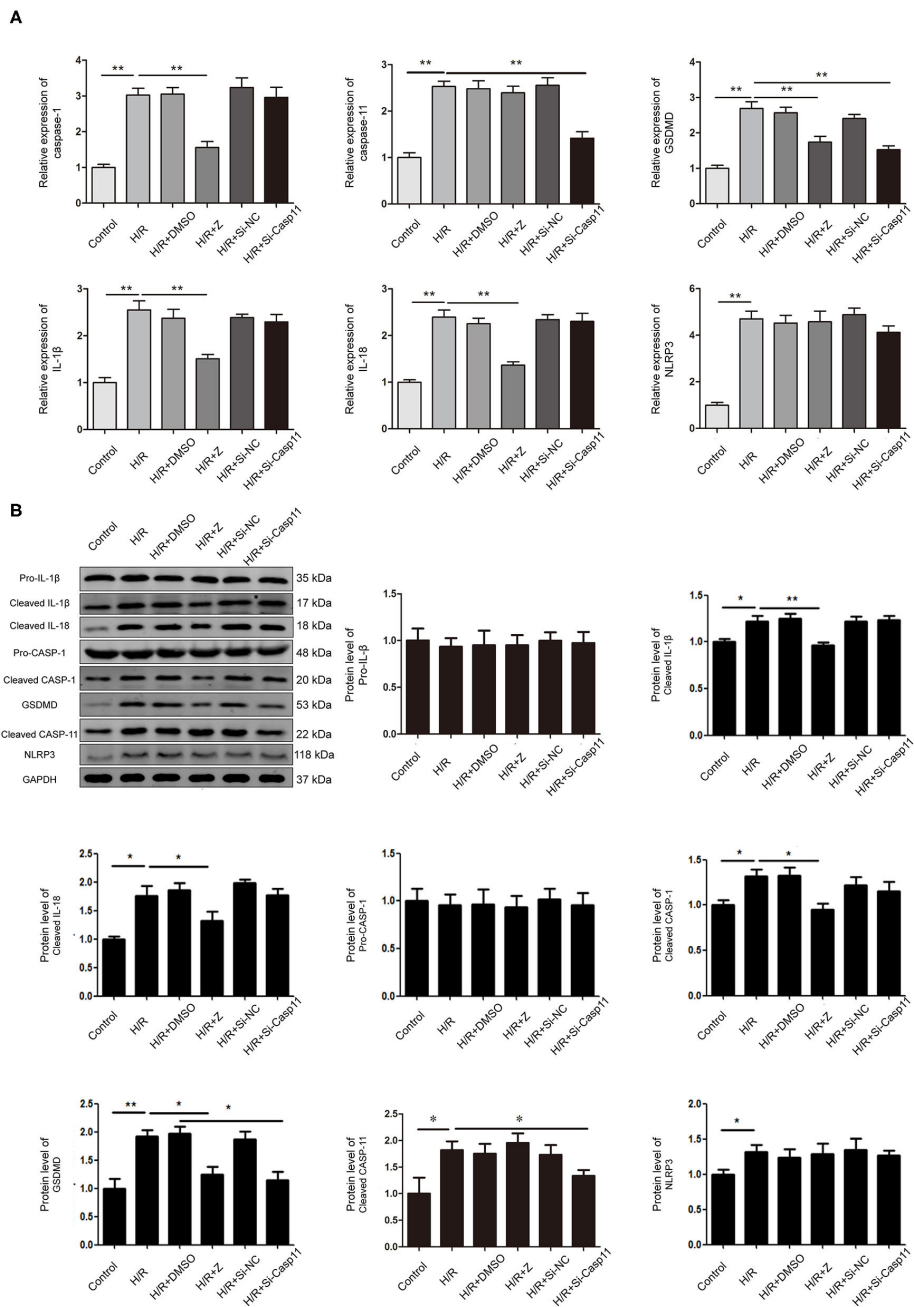
Effect of Z-YVAD-FMK and si-caspase-11 on protein and mRNA expression in the NRK-52E cell culture model. **(A)** mRNA levels of NLRP3, caspase-1, caspase-11, GSDMD, IL-1β, and IL-18 in NRK-52E cells after H/R. **(B)** Protein expression and statistical analysis of NLRP3, pro-caspase-1, cleaved-caspase-1, cleaved-caspase-11, GSDMD, pro-IL-1β, cleaved-IL-1β and cleaved-IL-18 in NRK-52E cells after caspase-1 or caspase-11 inhibition. *p < 0.05, **p < 0.01. Z = Z-YVAD-FMK; H/R, hypoxia/reoxygenation; DMSO, dimethyl sulfoxide; Si-casp11, small interfering ribonucleic acid caspase-11; Si-NC, si-caspase-11 negative control; GSDMD, gasdermin D; GAPDH, glyceraldehyde-3-phosphate dehydrogenase.

RT-qPCR and western blotting results showed that the expression of NLRP3, caspase-1, caspase-11, IL-1β, IL-18, and GSDMD in NRK-52E cells was upregulated under H/R conditions (Figures 6A,B). Protein and mRNA expression of IL-1β, IL-18, caspase-1, and GSDMD was lower in the Z-YVAD-FMK group than in the H/R group (Figures 6A,B); however, there was no significant change in NLRP3 or caspase-11 expression (Figures 6A,B). Further, mRNA and protein expression of caspase-11 were significantly lower in the siRNA group than in the H/R group (Figures 6A,B); mRNA expression of GSDMD was significantly lower in the siRNA group than in the H/R group (Figure 6A); However, there was no significant change in protein and mRNA expression of NLRP3, caspase-1, IL-1β, and IL-18 in the siRNA group (Figures 6A,B).

## Discussion

Changes in blood circulation in brain-dead organ donors can lead to severe ischemia-reperfusion injury, resulting in acute tubular necrosis and delayed organ function after kidney transplantation (27, 28). During BD, an inflammatory storm occurs, which causes drastic inflammatory changes in the donor organ before transplantation (29, 30).

Studies support the role of apoptosis in acute kidney injury (31, 32). Proximal tubular epithelial cells are susceptible to apoptosis, and damage to this region results in organ failure (9). However, preventing apoptosis alone cannot significantly improve renal function after transplantation; therefore, we aimed to explore the mechanism of pyroptosis in brain-dead donors. Here, our results suggested that pyroptosis was induced in kidney tissues after BD, and Z-YVAD-FMK treatment effectively improved the renal function and reduced renal injury in a brain-dead rat model thereafter.

We examined the expression of classical and non-classical pathway-related molecules in a brain-dead rat model. Caspase-1/11 belong to the proinflammatory caspase subfamily and play key roles in immune response-related signaling. Mice with caspase-1/11 gene knockout are more tolerant to *Escherichia coli-*induced septic shock than those lacking caspase-1 and IL-1β, suggesting that caspase-1/11 associated pathways act together in mice along with septic shock. Initially, caspase-1 and caspase-11 were thought to be associated with independent pathways; however, later, they were discovered as part of a complex regulatory network with mutual correlation and interaction (33, 34). Here, we found that both caspase-1 and caspase-11 were increased in BD rats and associated with BD-induced kidney injury.

Cao et al. (35) confirmed that NLRP3 inflammasome activation mediated blood-brain barrier dysfunction in cerebral ischemia, and inhibition of the same reduced blood-brain barrier injury after ischemia (35). In our BD model, expression of NLRP3 in the kidney was significantly increased, suggesting that it was one of the main receptors associated with inflammasome formation and initiation of the canonical pyroptotic pathway. NLRP3 promotes the activation of caspase-1, induces release of IL-1β and IL-18, and leads to renal injury, confirming that certain stimulating factors induced by BD activate NLRP3 in rats and promote occurrence of canonical pyroptosis thereafter (36).

GSDMD serves as a key executioner of pyroptosis in experimental cerebral ischemia and reperfusion model both in *vivo* and in *vitro* (21). Here, a significant increase in GSDMD was detected in the kidneys of BD rats, demonstrating that GSDMD cleavage was necessary and sufficient for inflammatory caspase activation-induced pyroptosis. Both mRNA and protein expression of GSDMD in the BD + Z-YVAD-FMK group were significantly decreased, indicating that caspase-1 expression was inhibited by Z-YVAD-FMK, and the expression of GSDMD correspondingly decreased.

Pyroptosis is involved in the cryopreservation and auto-transplantation of mouse ovarian tissues, and its inhibition can improve ovarian graft function (37). In our study, Z-YVAD-FMK effectively protected renal function in BD rats. In the H/R model, we verified that caspase-1, caspase-11, and GSDMD were significantly upregulated; whereas, addition of Z-YVAD-FMK abrogated this effect. NRK-52E cell viability decreased significantly in the H/R environment and Z-YVAD-FMK treatment increased the cell viability significantly (Figure 5F).

Previous studies on pyroptosis (38) have mainly focused on the role of caspase-1 in the canonical pathway; here, we focused on whether caspase-11-mediated pyroptosis could be involved in BD-related organ injury. The level of caspase-11 in brain-dead kidney tissues was significantly increased, as determined by IHC, mRNA and protein expression. However, caspase-11-mediated atypical pyroptotic pathway was not affected by the caspase-1 inhibitor. Caspase-11 was knocked down by an siRNA, and the results revealed that H/R activated both canonical and non-canonical pyroptosis. Z-YVAD-FMK inhibited the expression of IL-1β and IL-18 (Figures 6A,B), thereby indicating the increased protective effect of Z-YVAD-FMK on cell viability after H/R. However, caspase-11 knockdown did not exhibit a protective effect on cell viability after H/R. Therefore, we concluded that canonical pyroptosis was the major pathway that affected H/R injury in NRK-52E cells.

This study has a few limitations. This is a rat model and the results are therefore not automatically transferable to humans. The sample size (especially of the control groups) is naturally small in animal experiments. We did not explore the role of pyroptosis in kidney injury beyond 6 h. Thus, further studies are required to understand the potential mechanisms of action. In addition, the solvent of Z-YVAD-FMK in this was DMSO and limited the further studies in human.

In summary, our study shows that pyroptosis promote inflammation and induce kidney injury after brain death in rats. Z-YVAD-FMK reduced the inflammation and cell injury in rats and cell experiment. Pyroptosis could be considered as a therapeutic target for BD-induced kidney injury.

## Data Availability Statement

The original contributions presented in the study are included in the article/supplementary materials, further inquiries can be directed to the corresponding author/s.

## Ethics Statement

The animal study was reviewed and approved by Ethics Committee of the First Affiliated Hospital of Zhengzhou University (No. 2019-KY-019).

## Author Contributions

WL, DY, JS, PW, SC, WG, and SZ conceived and planned the experiments and contributed to the interpretation of the results. WL, DY, JS, JZ, ZW, BH, and XS carried out the experiments. JZ, ZW, BH, XS, and SC contributed to sample preparation. WL, DY, JS, WG, and SZ processed the experimental data, performed the analysis, drafted the manuscript and designed the figures. WL took the lead in writing the manuscript. All authors provided critical feedback and helped shape the research, analysis, and manuscript.

## Funding

This work was supported by the National Natural Science Foundation of China (No. 81971881) and Medical Science and Technology Project of the Henan Province Health Commission (SB201901045).

## Conflict of Interest

The authors declare that the research was conducted in the absence of any commercial or financial relationships that could be construed as a potential conflict of interest.

## Publisher's Note

All claims expressed in this article are solely those of the authors and do not necessarily represent those of their affiliated organizations, or those of the publisher, the editors and the reviewers. Any product that may be evaluated in this article, or claim that may be made by its manufacturer, is not guaranteed or endorsed by the publisher.

## Abbreviations

BD: brain death
DMSO: dimethyl sulfoxide
NLRP3: the nod-like receptor family pyrin domain-containing 3
GSDMD: gasdermin D
IHC: immunohistochemistry
H/R: hypoxia/reoxygenation
NRK52E: renal tubular epithelial cells
GAPDH: glyceraldehyde-3-phosphate dehydrogenase.
